# Serum lactate dehydrogenase predicts prognosis and correlates with systemic inflammatory response in patients with advanced pancreatic cancer after gemcitabine-based chemotherapy

**DOI:** 10.1038/srep45194

**Published:** 2017-03-27

**Authors:** Shu-Lin Yu, Li-Tao Xu, Qi Qi, Ya-Wen Geng, Hao Chen, Zhi-Qiang Meng, Peng Wang, Zhen Chen

**Affiliations:** 1 Department of Integrative Oncology, Fudan University Shanghai Cancer Center, 270 Dong An Road, Shanghai 200032, China; 2 Department of Oncology, Shanghai Medical College, Fudan University, 130 Dong An Road, Shanghai 200032, China

## Abstract

Serum lactate dehydrogenase (LDH) concentrations correlate with tumor progression and poor outcome. We evaluated the predictive value of serum LDH level for overall survival (OS) of patients with advanced pancreatic cancer after gemcitabine-based chemotherapy. We retrospectively enrolled 364 patients with locally advanced or metastatic pancreatic adenocarcinoma who were then allocated to training (n = 139) and validation cohorts (n = 225). We evaluated the association between serum LDH levels and OS as well as with markers of systemic inflammation, including neutrophil/lymphocyte ratio (NLR), platelet/lymphocyte ratio (PLR) and lymphocyte/monocyte ratio (LMR). Kaplan–Meier analyses revealed that low serum LDH levels in the training cohort significantly correlated with longer OS. Multivariate analysis identified the serum LDH levels as an independent prognostic predictor of OS (p = 0.005). Serum LDH levels correlated positively with NLR and PLR and correlated negatively with LMR. Similar results were obtained for the validation cohort, except that multivariate analysis identified the serum LDH level as a significant prognostic predictor and only a statistical trend for OS (p = 0.059). We conclude that serum LDH levels were associated with the systemic inflammatory response and served as a significant prognostic predictor of OS. Serum LDH levels predicted OS in patients with advanced pancreatic cancer after gemcitabine-based palliative chemotherapy.

Pancreatic cancer is the most lethal commonly occurring cancer, because it is usually diagnosed at an advanced stage and is resistant to therapy^1^. Moreover, pancreatic cancer is the fourth leading cause of cancer death in Western countries and is projected as the second leading cause within a decade^2^. Patients with pancreatic cancer survive for a median of 6 months, and 5-year survival is <5%, despite 50 years of research and therapeutic advances^3^. Patients’ continuing poor prognosis may be attributed to the invasive phenotype and complex mechanisms of chemoresistance of pancreatic cancers as well as the key involvement of hypoxia in pancreatic ductal adenocarcinoma (PDAC). Therefore, there is an urgent requirement to identify molecular markers that can guide the implementation of optimal therapeutic strategies and to indicate patients’ prognoses.

Clinical tumour markers help diagnose, determine prognosis, and assess therapeutic responses of patients with gastroenterological cancers. Examples include carbohydrate antigen (CA) 19-9, carcinoembryonic antigen (CEA), CA242, CA724, CA50, CA125, CA153, α-fetoprotein (AFP) and lactate dehydrogenase (LDH)^4^,^5^,^6^. Serum levels of CA19-9, CEA, CA50, SPan-1, peanut agglutinin, Du-PAN-2, AFP, tissue polypeptide antigen (TPA) and pancreatic oncofoetal antigen generally increase in patients with pancreatic cancer^7^. Although these markers are useful for monitoring pathologically diagnosed disease, their levels increase in patients with benign pancreatic disease (BPD)^8^,^9^. For example, CA19-9 is routinely used as a marker for pancreatic cancer and may reflect tumor burden. However, serum CA19-9 levels are higher in patients with PDAC compared with those of healthy controls, and they are significantly increased in the sera of patients with BPD^10^. Early detection of pancreatic cancer is difficult, mainly because of the absence of specific serum biomarkers and the retroperitoneal location of the pancreas.

Serum LDH levels are associated with tumor expression and poor outcomes, and LDH assays are relatively inexpensive and easy to perform. Therefore, we focused our attention on the prognostic value of serum LDH levels in patients with advanced pancreatic cancer after they were administered gemcitabine-based palliative chemotherapy. LDH, which is a key enzyme in glycolysis, is required for the anaerobic conversion of pyruvate to lactate^11^,^12^. LDH levels are regulated by the PI3K/AKT/mTOR pathway, the MYC oncogenic transcription factor, tumor hypoxia and necrosis^13^,^14^. Under physiological conditions, serum LDH concentrations range from 120–250 IU/mL and increase in patients with tumours, liver disease or cardiopathy. LDH levels correlate with tumour burden and may reflect tumour growth and invasive potential^15^. Moreover, LDH levels serve as a prognostic marker of various malignancies such as colorectal and breast cancer, lymphoma, melanoma, renal cell carcinoma and germ-cell tumours^16^,^17^,^18^,^19^,^20^,^21^,^22^,^23^. However, the prognostic value of serum LDH levels in patients with advanced pancreatic cancer after they are administered gemcitabine-based palliative chemotherapy is unknown.

Cancer-associated inflammation may serve as the seventh hallmark of cancer, which affects a patient’s response to chemotherapeutic agents and survival. The biological link between hypoxia, LDH levels and the tumour-driven angiogenesis pathway may be explain the abnormal activation of the hypoxia inducible factor 1 (HIF-1)^24^. Further, evidence indicates that HIF-1 promotes inflammation and fibrosis in patients with PDAC, indicating a correlation between hypoxia and systemic inflammation. Moreover, serum LDH levels serve as an indirect marker of tumour hypoxia. However, the correlation between the systemic inflammation and serum LDH level has not been evaluated in patients with pancreatic cancer after they are administered gemcitabine-based palliative chemotherapy. To address these questions, here we evaluated the independent prognostic significance of serum LDH levels and their potential associations with cancer-specific survival and the systemic inflammationin a large cohort of such patients.

## Results

### Patients’ characteristics

We enrolled 364 consecutive patients with the characteristics as follows: 229 (62.9%) were men, 177 (48.6%) had CA19-9 concentrations ≥1000 IU/mL and 92 (25.3%) were diagnosed with stage III pancreatic cancer. The tumour was located in the pancreatic head in 143 patients (39.3%) and in the body/tail in 221 (60.7%). All patients received gemcitabine-based palliative chemotherapy. The median number of times that patients received gemcitabine-based interventional therapy or conventional systemic venous chemotherapy (gemcitabine monotherapy or combinations such as gemcitabine plus cisplatin/oxaliplatin or gemcitabine plus albumin-bound paclitaxel) were 2.04 and 3.26, respectively, in the training cohort. The median number of times that patients in the validation cohort received these respective therapies were 2.43 and 3.91,respectively. The total numbers of treatments were calculated as the interval between the date of the first treatment and death (or the last observation point taken).The first 139 patients were assigned to the training cohort, and the remaining 225 were assigned to the validation cohort. The clinicopathological characteristics of the patients are summarized in Table 1.

### Value of serum LDH levels for predicting OS

**Figure 1:**
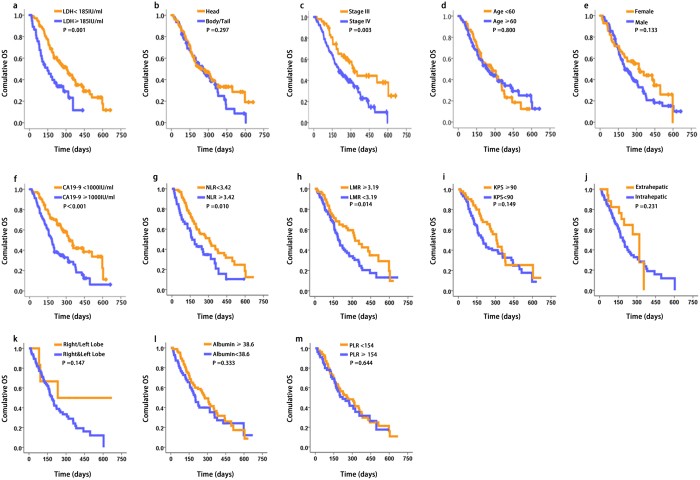
Association of serum LDH levels and OS of patients with advanced pancreatic cancer in the training cohort (n = 139). Kaplan–Meier analysis of OS of patients in the training cohort. The median LDH concentration was 185 IU/mL and was selected as the cut-off between high and a low LDH levels. The p-value was determined using the log-rank test and Kaplan–Meir curves were generated as functions of the variables as follows: (**a**) LDH levels, (**b**) tumour location, (**c**) disease stage, (**d**) age, (**e**) sex, (**f**) CA19-9 levels, (**g**) NLR, (**h**) LMR, (**i**) KPS, (**j**) metastasis, (**k**) location of intrahepatic metastasis, (**l**) albumin concentrations and (**m**) PLR.

Kaplan–Meier analysis of the OS of patients in the training cohort revealed that the serum level of LDH, tumour stage (III vs. IV), level of CA19-9, NLR and MLR significantly affected prognosis (Fig. 1a,c,f–h). In contrast, there was not a significant correlation between the serum level of LDH and tumour location, age, sex, Karnofsky performance status (KPS), metastasis (intrahepatic or extrahepatic), location of intrahepatic metastasis (right, left or both lobes), albumin or PLR (Fig. 1b,d,e,i–m). Moreover, low serum LDH levels (<185 IU/mL) significantly correlated with longer OS of patients in the training cohort (148 days and 308 days for patients with high or low LDH levels, respectively, 95% CI: 174.7–333.3; log rank, 10.504; p = 0.001) (Fig. 1a).

**Figure 2:**
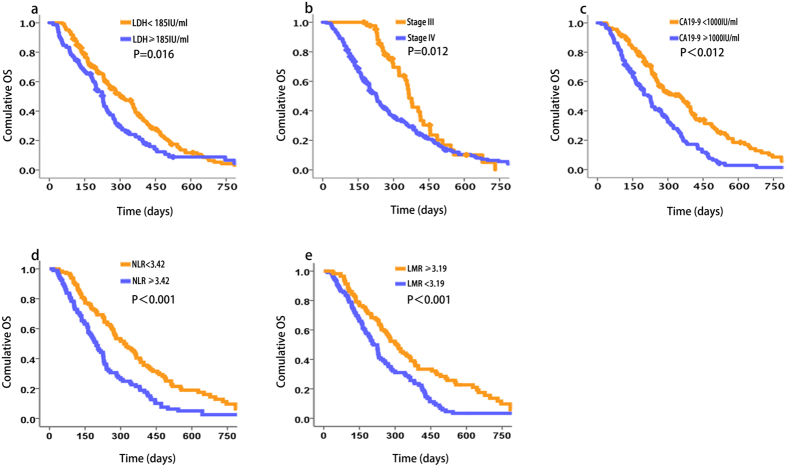
Association of serum LDH levels and OS of patients with advanced pancreatic cancer in the validation cohort (n = 225). Kaplan–Meier analysis of OS of patients in the validation cohort. The median LDH concentration of samples was 185 IU/mL and was selected as the cut-off between high and low LDH levels. The p-values were determined using the log-rank test. Kaplan–Meir curves were generated as functions of the variables as follows: (**a**) LDH levels, (**b**) disease stage, (**c**) CA19-9 concentrations, (**d**) NLR and (**e**) LMR.

We found that the level of serum LDH, stage (III vs. IV), CA19-9, NLR and MLR affected prognosis (Fig. 2a–e). In contrast, there was not a significant correlation between the serum level of LDH and tumour location, age, sex, KPS, metastasis (intrahepatic or extrahepatic), location of intrahepatic metastasis (right, left, or both lobes), albumin or PLR, similar to the findings for the test cohort. The prognostic value of LDH was confirmed in the independent validation cohort (OS 226 days and 301 days for patients with high (≥185 IU/mL) and low LDH levels, respectively, 95% CI: 283.4.7–342.7; log rank, 5.793; p = 0.016) (Fig. 2a). Further, OS was independent of tumour stage (III vs. IV). Together, the results suggest that serum LDH levels were significantly associated with OS of patients with advanced pancreatic cancer.

### Univariate and multivariate analysis of prognostic factors in the training and validation cohorts

In the training cohort, 93/139 patients (67%) died during the observation period, and 46 (33%) were alive after a median follow-up of 78 months. The median OS of the training population was 254 days (95% CI: 174.7–333.3). Univariate analysis was performed to determine the prognostic value of serum LDH level and other clinical variables for OS of the training cohort (Table 2). Poor OS was predicted by tumour stage (III vs. IV), CA 19-9 (<1000 vs. ≥1000 IU/mL), serum LDH levels (≥185 IU/mL vs. <185 IU/mL), NLR (<3.42 vs. ≥3.42) and LMR(<3.19 vs. ≥3.19) (p = 0.004, p <0.001, p = 0.002, p < 0.001 and p = 0.001, respectively). These five variables were included in the multivariate analysis using Cox proportional hazards regression. Tumour location, age, sex, KPS (<90 vs. ≥90), albumin (<38.6 g/L vs. ≥38.6 g/L), metastasis (intrahepatic or extrahepatic), location of intrahepatic metastasis (right, left or both lobes) and PLR (<154 vs. ≥154) were not significantly associated with OS (all p > 0.05). Multivariate analysis identified serum LDH levels as an independent prognostic predictor of OS (HR = 1,981; 95% CI: 1.226–3.200; p = 0.005) (Table 2).

In the validation cohort, 190 of the 225 patients (84%) died during the observation period, and 35 (16%) were alive after a median follow-up of 78 months. The median OS of the training population was 260 days (95% CI: 225.8–294.2). Univariate analysis revealed that age (<60 years vs. ≥60 years), tumour stage (III vs. IV), CA 19-9 (<1000 vs. ≥1000 IU/mL), serum LDH levels (≥185 IU/mL vs. <185 IU/mL), NLR (<3.42 vs. ≥3.42) and LMR (<3.19 vs. ≥3.19) predicted poor OS (p = 0.056, p = 0.013, p <0.001, p = 0.016, p = 0.004 and p = 0.001, respectively). These six variables were included in the multivariate analysis using Cox proportional hazards regression. Similarly, tumor location, sex, KPS (<90 vs. ≥90), albumin (<38.6 g/L vs. ≥38.6 g/L), metastasis (intrahepatic or extrahepatic), location of intrahepatic metastasis (right, left or both lobes) and PLR (<154 vs. ≥154) were not significantly associated with OS (all p > 0.05). Multivariate analysis (Table 3) confirmed that serum LDH levels have prognostic value for OS (HR = 0.747; 95% CI: 0.552–1.012; p = 0.059).

### Association of serum LDH levels with markers of systemic inflammation

**Figure 3:**
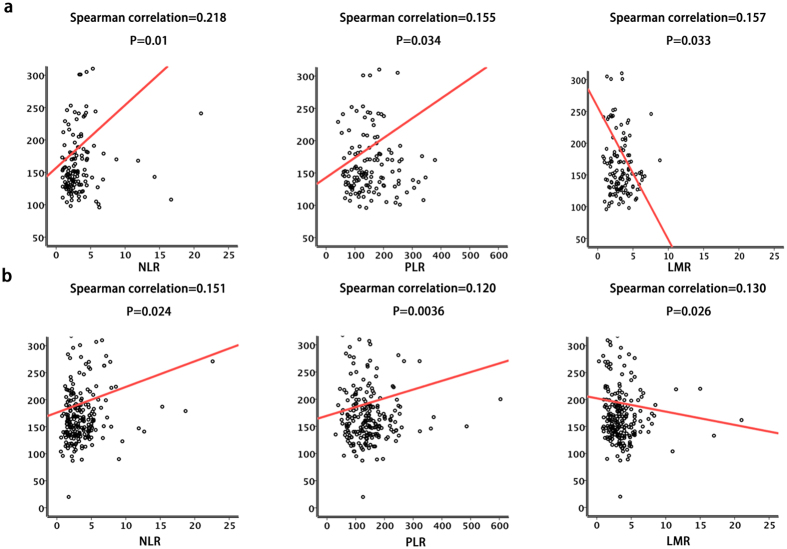
Serum LDH levels were associated with markers of systemic inflammation. Serum LDH levels significantly correlated with NLR, PLR and MLR levels in the (**a**) training and (**b**) validation cohorts (Spearman correlation).

Systemic inflammatory response markers such as NLR, PLR and MLR serve as prognostic predictors of OS of patients with certain cancers, including pancreatic cancer. Further, hypoxia and systemic inflammation are associated with the advanced stages of pancreatic cancer, and serum LDH levels serve as an indirect marker of tumour hypoxia. Therefore, we evaluated the relationship between serum LDH levels and markers of systemic inflammation in both cohorts (Table 1). Tests for normality (Kolmogorov-Smirnov) indicated that the data represented a continuous parameter that did not meet the assumptions of the test for normality (p < 0.001), except for LMR in the training cohort (p = 0.2). Therefore, we used Spearman’s non-parametric test. In the training cohort, serum LDH levels positively correlated with NLR (r = 0.218, P = 0.01) and PLR (r = 0.155, P = 0.034) but negatively correlated with LMR (r = −0.157, P = 0.033). These results were further confirmed in the validation cohort of 225 cases. Thus, serum LDH levels positively correlated with NLR (r = 0.151, P = 0.0024) and PLR (r = 0.120, P = 0.036) but negatively correlated with LMR (r = −0.130, P = 0.026) (Fig. 3a,b). We concluded therefore that serum LDH levels were associated with the systemic inflammatory response in advanced pancreatic cancer after gemcitabine-based palliative chemotherapy.

## Discussion

Here we investigated the correlation between LDH levels and the prognosis of patients with advanced pancreatic cancer after they were administered gemcitabine-based palliative chemotherapy. The results show that high vs. low serum LDH levels significantly correlated with shorter OS. Further, serum LDH levels correlated positively with NLR and PLR and negatively with LMR.

The oxidoreductase LDH, which converts pyruvate to lactate when oxygen is absent or in short supply, plays a crucial role in the metabolism of cancer cells^25^. Tetrameric human LDH comprises three different monomeric subunits LDH-A, LDH-B and LDH-C. LDH-A is overexpressed in hypoxic carcinomas as well as metastatic cancer cells, and its levels correlate with tumour viability^26^. In many tumour types, serum LDH levels serve as an indirect marker of tumour hypoxia, neo-angiogenesis and worse prognosis. PDACs are characterized by a dense desmoplastic stroma and a sparse vascularization that limit the availability of oxygen and nutrients. This tissue architecture induces severe hypoxic stress in tumour cells, which then become resistant to chemotherapy and develop increased invasiveness and metastatic potential^27^. Together, these factors led us to explore the relationship between LDH levels and the prognosis of patients with advanced pancreatic cancer.

Increased LDH levels are present in patients with malignancies, including pancreatic cancer, and elevated LDH levels serve as a prognostic marker for numerous human malignancies^16^,^17^,^18^,^19^,^20^,^21^,^22^,^23^. Further, LDH levels can guide the treatment of pancreatic cancer. For example, the inhibition by oxamic acid of LDH production by cancer cell lines potentiates the antiproliferative activity of tyrosine kinase inhibitors such as sorafenib^28^, Moreover, patients with high pretreatment levels of LDH may represent optimal candidates for inclusion in clinical trials that employ a multimodality treatment approach (e.g. transcatheter arterial chemoembolization and inhibitors of vascular endothelial growth factor signaling) to improve time to progression and OS^29^.

Here we found a significant association between serum LDH levels and OS in 346 patients with advanced pancreatic cancer after they were administered gemcitabine-based palliative chemotherapy. Specifically, low serum LDH levels significantly correlated with longer OS. Only tumour stage and CA19-9 are widely used for predicting prognosis of patients with advanced pancreatic cancer^30^. CA19-9 is the most commonly used marker for pancreatic cancer to evaluate prognosis^31^. Evidence suggests that other serum tumour markers such as LDH may have prognostic relevance for patients with advanced pancreatic cancer^32^,^33^. Several preclinical studies provide evidence that the pathogenesis and progression of advanced pancreatic cancer involves a hypoxic tumour microenvironment that is associated with high tumour volume, high tumour LDH expression, and frequent inflammation.

Inflammation is the seventh hallmark of cancer that can affect a patient’s response to chemotherapeutic agents and survival. Evidence indicates a correlation between hypoxia and systemic inflammation. We show here that serum LDH levels correlated positively with NLR and PLR and negatively with LMR. The modified Glasgow prognostic score and C-reactive protein (CRP) levels are valuable and commonly used markers of systemic inflammation, which correlate with the prognosis of numerous human cancers, including pancreatic cancer. Further, interleukin-6 (IL-6) is an important predictor of OS. However, we were unable to evaluate the clinical significance of IL-6 levels here, because assays for CRP and IL-6 were not routinely performed in our clinic. Because of this limitation, we will evaluate the prognostic value of other common markers of systemic inflammation, including CRP and IL-6, in patients with pancreatic cancer and their correlation with serum LDH levels in future studies.

In conclusion, we found that serum LDH had prognostic value and was associated with systemic inflammation in patients with advanced pancreatic cancer after they were administered gemcitabine-based palliative chemotherapy. To accurately determine the roles and clinical significance of candidate biomarkers for improving the management of patients with pancreatic cancer, large prospective studies are required that employ uniform disease staging and standardized cut-off levels of biomarkers.

## Materials and Methods

### Patients’ characteristics and clinical features

All patients, their relatives or both provided written informed consent for their clinical and pathological information to be used for research and stored in the hospital’s database. The Ethics Committee of Fudan University Shanghai Cancer Center (FUSCC) approved the methods and experimental protocols used in the present study, which were performed in accordance with the ethical standards of our institutional research committee and the tenants of the 1964 Declaration of Helsinki and its later amendments or comparable ethical standards. Methods (Ethics Number: 050432-4-1212B) were performed in accordance with the approved guidelines. We retrospectively recruited 364 patients with histologically or cytologically confirmed locally advanced or metastatic pancreatic adenocarcinoma who were treated at FUSCC between January 2010 and December 2013. The criteria for locally advanced disease included tumour invasion of the celiac trunk, superior mesenteric artery or both, corresponding to stage III pancreatic cancer according to the International Union Against Cancer TNM Classification of Malignant Tumors (Sixth Edition). Dynamic Contrast-enhanced abdominal computed tomography (CT), magnetic resonance imaging (MRI) and MR-cholangiopancreatography were used for determining the TNM stage.

### Serum LDH assay

Serum LDH was assayed during routine workups to exclude liver-function disorders before diagnostic interventions or cancer treatment, and these levels were subjected to statistical analyses. LDH was measured in sera prepared from peripheral venous blood collected on the day of hospital admission using a method described by the International Federation of Clinical Chemistry and Laboratory Medicine. The median LDH concentration was 185 IU/mL and was selected as the cut-off between a high and low LDH levels.

### Laboratory measurements

Routine laboratory measurements, including counts of white blood cells, neutrophils, lymphocytes, monocytes and platelets as well as albumin concentrations were performed before diagnostic intervention or treatment. The neutrophil/lymphocyte ratio (NLR), platelet/lymphocyte ratio (PLR) and lymphocyte/monocyte ratio (LMR) were calculated. The median NLR, PLR, MLR values and albumin levels of 139 samples were 3.42, 154, 3.19 and 38.6 (g/L), respectively, and were selected as the cut-off values.

### Statistical analysis

Patients’ characteristics before diagnostic intervention or cancer treatment are reported using descriptive statistics. The χ^2^ or Fisher’s exact tests were used to compare quantitative outcomes between groups. Overall survival (OS) was defined as the interval between a definitive diagnosis and death or last follow-up. Survival curves were calculated using the Kaplan–Meier method, and the log-rank test was used to assess the significance of differences in survival. Univariate and multivariate analyses were performed using a Cox proportional hazards regression model as implemented in SPSS version 21.0 (IBM Corp., Armonk, NY, USA). Variables with p < 0.05 determined using univariate analysis were included as candidates in a multivariate Cox regression model (conditional backward selection). Hazard ratios (HRs) estimated from the Cox analysis are reported as relative risk (RR) with corresponding 95% confidence intervals (CIs). The correlation tests used a Spearman’s non-parametric test, and p < 0.05 was applied throughout to indicate a significant difference.

### Ethics Statement

All patients and/or their relatives provided a written informed consent for their clinical and pathological information to be used for research and stored in the hospital database; this study, methods and experimental protocols were approved by the Ethical Committee of FUSCC (Fudan University Shanghai Cancer Center). All the procedures performed in our study were in accordance with the ethical standards of our institutional research committee and the 1964 Helsinki declaration and its later amendments or comparable ethical standards. Methods(Ethics Number:050432-4-1212B) were carried out in accordance with the approved guidelines.

## Additional Information

**How to cite this article:** Yu, S.-L. *et al*. Serum lactate dehydrogenase predicts prognosis and correlates with systemic inflammatory response in patients with advanced pancreatic cancer after gemcitabine-based chemotherapy. *Sci. Rep.* **7**, 45194; doi: 10.1038/srep45194 (2017).

**Publisher's note:** Springer Nature remains neutral with regard to jurisdictional claims in published maps and institutional affiliations.

## Figures and Tables

**Table 1:** Clinical characteristics of patients in the training and validation cohorts.

Variables	Value	Training cohort (n = 139)			Validation cohort (n = 225)		
		LDH ≥ 185	LDH < 185	p-value^*^	LDH ≥ 185	LDH < 185	p-value^*^
Age (years)	<60	15	39	0.226	33	45	0.636
	≥60	22	63		63	84	
Sex	Male	19	64	0.805	49	97	0.937
	Female	18	38		29	50	
Stage	III	8	40	0.054	12	32	0.251
	IV	29	62		66	115	
CA 19-9 (IU/ml)	≥1000	20	48	0.335	40	69	0.138
	<1000	17	54		38	78	
Location	Head	19	43	0.466	23	55	0.535
	Body/tail	18	59		58	89	
PLR	≥154	15	40	0.888	25	49	0.846
	<154	22	62		53	98	
NLR	≥3.42	19	27	0.006	38	56	0.124
	<3.42	18	75		40	91	
LMR	≥3.19	16	51	0.481	35	81	0.144
	<3.19	21	51		43	66	
KPS	≥90	8	44	0.028	51	86	0.314
	<90	29	58		51	61	
Metastasis	Intrahepatic	26	48	0.163	58	97	0.01
	Extrahepatic	3	14		3	23	
Location of intrahepatic metastasis	Left Lobe	0	0	0.323	1	5	0.249
	Right Lobe	1	5		5	12	
	Left&Right Lobe	25	43		51	81	
Albumin (g/L)	≥38.6	18	66	0.087	58	106	0.718
	<38.6	19	36		20	41	

*Pearson χ^2^test.

LDH, lactate dehydrogenase; KPS, Karnofsky performance status.

**Table 2:** Univariate and multivariate Cox regression analyses of LDH and OS of patients with advanced pancreatic cancer in the training cohort.

Variable	Patients (n)	HR (95% CI)	p-value^*^
**Univariate analysis**			
Sex (male vs. female)	83 vs. 56	1.386 (0.903–2.129)	0.135
Age (<60 vs. ≥60)	54 vs. 85	0.947(0.623–1.441)	0.8
Stage (III vs. IV)	48 vs. 91	1.993 (1.244–3.194)	0.004
Location (head vs. body/tail)	62 vs. 77	0.805 (0.535–1.21)	0.299
CA19-9 (<1000 vs. ≥1000 IU/mL)	71 vs. 68	2.037 (1.344–3.088)	<0.001
LDH (<185 vs. ≥185 IU/mL)	102 vs. 37	2.108 (1.329–3.346)	0.002
KPS (<90 vs. ≥90)	87 vs. 52	1.374 (0.891–2.118)	0.151
Metastasis (Intrahepatic vs. Extrahepatic)	74 vs. 17	0.648 (0.316–1.327)	0.235
Location of intrahepatic metastasis (Left/Rirht Lobe vs. Left&Right Lobe)	6 vs. 68	2.335 (0.718–7.592)	0.159
Albumin (<38.6 vs. ≥38.6 g/L)	55 vs. 84	0.817 (0.541–1.232)	0.335
PLR (<154 vs. ≥154)	84 vs. 55	1.104 (0.725–1.683)	0.644
NLR (<3.42 vs. ≥3.42)	93 vs. 46	2.522 (1.633–3.894)	<0.001
LMR (<3.19 vs. ≥3.19)	72 vs. 67	0.497 (0.324–0.762)	0.001
**Multivariate analysis**			
Stage (III vs. IV)	48 vs. 91	1.623 (1.003–2.629)	0.049
CA19-9 (<1000 vs. ≥1000 IU/mL)	71 vs. 68	1.790 (1.159–2.764)	0.009
LDH (<185 vs. ≥185 IU/mL)	102 vs. 37	1.981 (1.226–3.200)	0.005
NLR (<3.42 vs. ≥3.42)	93 vs. 46	2.028(1.293–3.181)	0.002
LMR(<3.19 vs. ≥3.19)	72 vs. 67	0.883 (0.535–1.458)	0.627

*log rank χ^2^ test.

LDH, lactate dehydrogenase; KPS, Karnofsky performance status; HR, hazard ratio; CI, confidence interval.

**Table 3:** Univariate and multivariate Cox regression analyses of LDH levels and OS of patients with advanced pancreatic cancer in the validation cohort.

Variable	Patients (n)	HR (95% CI)	p-value*
**Univariate analysis**			
Sex (male vs. female)	146 vs. 79	0.754 (0.554–1.026)	0.073
Age (<60 vs. ≥60)	88 vs. 137	1.014 (1.000–1.029)	0.056
Stage (III vs. IV)	44 vs. 181	1.616 (1.107–2.36)	0.013
Location (head vs. body/tail)	81 vs. 144	0.97 (0.718–1.309)	0.84
CA19-9 (<1000 vs. ≥1000 IU/mL)	116 vs. 109	1.931 (1.44–2.588)	<0.001
LDH (<185 vs. ≥185 IU/mL)	147 vs. 78	0.694 (0.514–0.937)	0.016
KPS (<90 vs. ≥90)	137 vs. 88	1.052 (0.784–1.412)	0.735
Metastasis (Intrahepatic vs. Extrahepatic)	155 vs. 26	1.314 (0.846–2.039)	0.224
Location of intrahepatic metastasis (Left/Rirht Lobe vs. Left&Right Lobe)	23 vs. 132	1.072 (0.630–1.823)	0.799
Albumin (<38.6 vs. ≥38.6 g/L)	61 vs. 164	0.813 (0.590–1.120)	0.205
PLR (<154 vs. ≥154)	151 vs. 74	1.123 (0.826–1.527)	0.458
NLR (<3.42 vs. ≥3.42)	131 vs. 94	1.607 (1.197–2.159)	0.002
LMR (<3.19 vs. ≥3.19)	109 vs. 116	0.661 (0.493–0.886)	0.006
**Multivariate analysis**			
Age (<60 vs. ≥60)	88 vs. 137	0.934(0.694–1.258)	0.655
Stage (III vs. IV)	44 vs. 181	0.639 (0.431–0.947)	0.026
CA19-9 (<1000 vs. ≥1000 IU/mL)	116 vs. 109	0.626 (0.462–0.849)	0.003
LDH (<185 vs. ≥185 IU/mL)	147 vs. 78	0.747 (0.552–1.012)	0.059
NLR (<3.42 vs. ≥3.42)	131 vs. 94	0.777(0.534–1.131)	0.188
LMR(<3.19 vs. ≥3.19)	109 vs. 116	1.420(1.047–1.927)	0.024

*log rank χ^2^ test.

LDH, lactate dehydrogenase; KPS, Karnofsky performance status; HR, hazard ratio; CI, confidence interval.
